# The Cellular Stress and Cutaneous Manifestations in Renal Cell Carcinomas—A Narrative Review

**DOI:** 10.3390/jcm13133640

**Published:** 2024-06-21

**Authors:** Corina Daniela Ene, Ilinca Nicolae, Mircea Tampa, Simona Roxana Georgescu, Cosmin Ene, Clara Matei, Iulia Maria Teodora Leulescu, Cristina Iulia Mitran, Madalina Irina Mitran, Cristina Capusa

**Affiliations:** 1 Department of Nephrology, ‘Carol Davila’ University of Medicine and Pharmacy, 020021 Bucharest, Romania; corina.ene@umfcd.ro (C.D.E.); cristina.capusa@umfcd.ro (C.C.); 2 Department of Nephrology, ‘Carol Davila’ Nephrology Hospital, 010731 Bucharest, Romania; 3 Research Department, ‘Victor Babes’ Clinical Hospital for Infectious Diseases, 030303 Bucharest, Romania; drnicolaei@yahoo.ro; 4 Department of Dermatology, ‘Carol Davila’ University of Medicine and Pharmacy, 020021 Bucharest, Romania; matei_clara@yahoo.com; 5 Department of Dermatology, ‘Victor Babes’ Clinical Hospital for Infectious Diseases, 030303 Bucharest, Romania; iulialeulescu@gmail.com; 6 Department of Urology, ‘Carol Davila’ University of Medicine and Pharmacy, 020021 Bucharest, Romania; cosmin85_ene@yahoo.com; 7 Department of Urology, “Saint John” Clinical Emergency Hospital, 13 Vitan-Barzesti, 042122 Bucharest, Romania; 8 Department of Microbiology, ‘Carol Davila’ University of Medicine and Pharmacy, 020021 Bucharest, Romania; cristina.iulia.mitran@gmail.com (C.I.M.); madalina.irina.mitran@gmail.com (M.I.M.)

**Keywords:** renal cell carcinoma, cellular stress, secondary cutaneous manifestations

## Abstract

The carcinomas originating from the renal cortex are the most aggressive renal malignancies, with a high tendency for metastasis. Understanding the incidence of cutaneous manifestations caused by renal carcinomas is a challenge. In the first part, this article summarizes a series of factors that promote oncogenesis, invasiveness, and the ability of renal cell carcinoma (RCC) to develop secondary cutaneous manifestations. It is postulated that the cellular stress response is one of the leading causes of developing dermatological events induced by cancers located at distant sites. Furthermore, the paper provides an overview of cutaneous complications associated with renal cancer, categorized as malignant manifestations (metastases, synchronous or metachronous cutaneous malignancies associated with renal cancer), non-malignant indirect cutaneous manifestations associated with renal cancer, and treatment consequences. The data presented in this article suggest that recognizing certain cutaneous disorders could assist the physician in the early identification of renal neoplasms and could lead to a better prognosis.

## 1. Introduction

In recent decades, significant progress has been made in understanding the genetic and molecular mechanisms responsible for the development of renal tumors, which has led to improvements in patient care. Deregulated metabolism and oxidative stress pathways are commonly found in renal tumors and are essential factors influencing the progression of renal cell carcinoma (RCC) [1,2,3,4].

Renal cancer represented 2.2% of global cancer cases and 1.8% of cancer-related deaths in 2023, but it is estimated that this incidence will increase [1]. Renal cell carcinomas (RCCs), originating from the renal cortex, account for 80–85% of all primary renal neoplasms. Transitional cell carcinomas, originating in the renal pelvis, make up 8%. The most common subtypes of RCC are clear cell carcinomas (ccRCCs) (70%), papillary RCC (pRCC) (10%), and chromophobe RCC (chRCC) (5%), each with distinct genetic bases and phenotypes [1,2]. 

ccRCC is characterized in 90% of cases by von Hippel–Lindau (VHL) gene mutation. Papillary tumors can be divided into two distinct histological subtypes: papillary type I and papillary type II. Papillary type I includes tumors with MET mutations and chromosome-7 amplification. Papillary type II includes tumors with fumarate hydratase mutations and upregulation of the Nrf2-antioxidant response pathway [1,2].

ccRCC is an aggressive, highly vascularized, invasive cancer with late diagnosis, a low survival rate, a high tendency for metastasis, notable chemoresistance, a poor prognosis, and increased morbidity and mortality [1,3,4]. Due to these negative characteristics, 30% of RCC patients have metastatic disease at presentation, with metastases predominantly in the lungs (75%), bones (20%), liver (18%), and central nervous system (8%) [4,5,6]. Emerging molecular imaging techniques for the preoperative diagnosis of renal cell carcinoma are promising for reducing postoperative renal function loss. Improving the identification of aggressive tumors before surgery through advanced imaging techniques can decrease the number of surgeries, thereby reducing complications and morbidity. Thus, single photon emission computed tomography/computed tomography (SPECT/CT) and positron emission tomography/computed tomography (PET-CT) ensure a safe and rapid diagnosis of RCC [7].

Approximately 3% of RCC patients develop metastases and cutaneous paraneoplastic syndromes. Cutaneous manifestations can develop before the diagnosis of malignancy, but the metastatic form is more aggressive. It is linked to cellular stress in response to various insults and stressful conditions [8,9,10,11,12,13,14,15,16,17,18,19]. This cellular stress is strongly dysregulated in cancer. Cancer cells alter normal responses to oncogenic stress to redirect them towards supporting growth, even at the expense of organism integrity and homeostasis. The cellular stress response is a translational program of adaptive response activated by oncogenic stress, allowing a cell to survive in the presence of threatening factors, leading to cancer progression [8,20,21,22]. 

In this article, we will present the role of cellular stress in promoting RCC metastasis, describe the main cutaneous events generated by RCC, and discuss adverse cutaneous drug events in RCC patients.

## 2. Cellular stress in RCC Biology

**Figure 1:**
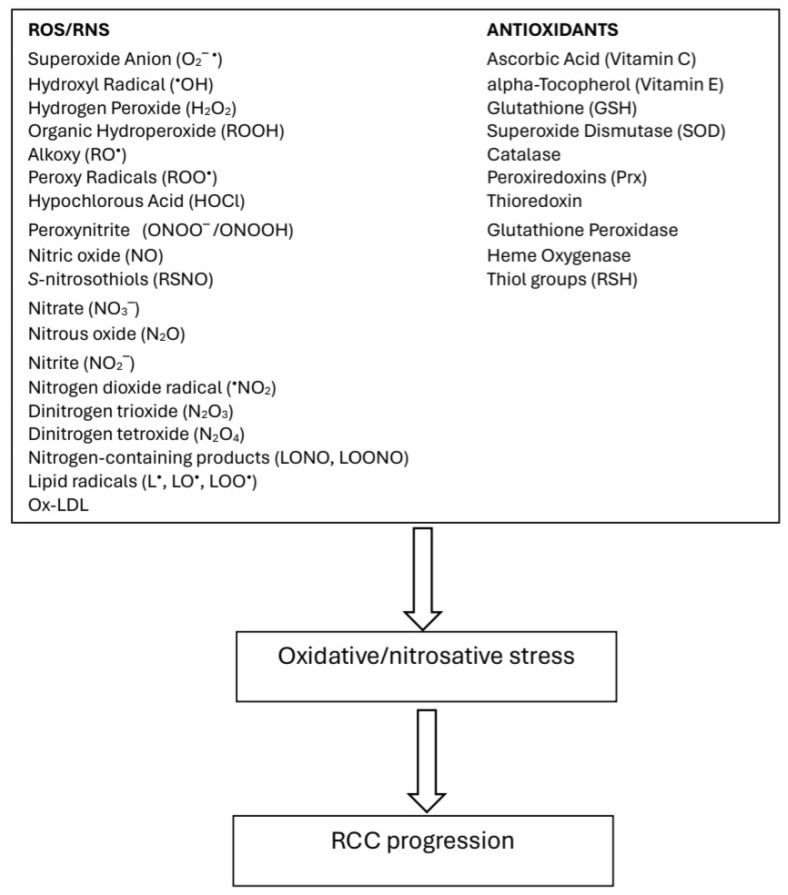
Nitrosative/oxidative stress in RCC.

Oxidative stress plays a significant role in tumorigenesis and tumor progression. Rapid cell proliferation, the energy needs of tumor cells, aerobic glycolysis, and inhibition of autophagy generate large amounts of reactive oxygen species (ROS) and reactive nitrogen species (RNS) in the body and, consequently, increase oxidative stress [1,2,3,4,12,13]. Elevated levels of ROS have been reported to affect tumor development by inducing cell apoptosis, deregulating tumor signaling, altering the tumor microenvironment, inducing immune evasion, promoting DNA mutations, and influencing therapeutic resistance [1,2,12,13,14,15]. An imbalance between endogenous oxidants and antioxidants results in oxidative stress, which contributes to the progression of RCC (Figure 1).

Potential inducers of cellular stress from within and outside cells are excessive amounts of oxidants [15]. Intense metabolic activity of tubular epithelial cells, glomerular cells, and activated macrophages is the main generator of oxygen reactive species (ROS) in human kidneys. 

In RCC, it has been documented that the response to cellular stress interferes with ROS production, angiogenesis, mitochondrial quality, tumor metabolism, inflammation, genetic instability, and the tumor microenvironment (TME) [8,10,20,21,22]. Low levels of chronic oxidative stress act as a driving force for the malignant transformation of renal epithelial cells [15,23,24]. An increase in ROS results in the activation of the NLRP3 inflammasome and the release of pro-inflammatory cytokines, which suppress immune surveillance, allowing tumor progression. Persistent stress increases susceptibility to cancer and enhances RCC’s capacity to develop secondary manifestations through a variety of mechanisms: tumor signaling, TME reorganization, immune evasion, invasion and metastasis, DNA mutations, angiogenesis, treatment response, and induction of cellular ferroptosis/apoptosis [13,25,26,27]. 

Based on bioinformatic analysis, several current studies have identified sets of genes related to oxidative stress (ORG). For instance, activating transcription factor 3 (ATF3) were correlated with RCC progression [28], genes ABCB1, AGER, E2F1, FOXM1, HADH, ISG15, KCNMA1, PLG, and TEK were correlated with the clinical outcome and immune status of ccRCC patients [27], genes UCN, PLG, FOXM1, and HRH2 were associated with the ccRCC prognosis [29], long non-coding RNAs (lncRNA) SPART-AS1, AL162586.1, LINC00944, LINC01550, HOXB-AS4, LINC02027, and DOCK9-DT were associated with ccRCC aggressiveness [30,31], mitochondrial genes ACAD11, ACADSB, BID, PYCR1, SLC25A27, and STAR were linked to the ccRCC prognosis [12], and genes ADAM8, CGN, EIF4EBP1, FOXM1, G6PC, HAMP, HTR2C, ITIH4, LTB4R, MMP3, PLG, PRKCG, SAA1, and VWF, and microRNAs related to the redox status and ccRCC progression [12], were found to be essential for the response to oxidative stress. Recently, a transcription factor for the oxidative stress response, broad complex-tramtrack-bric-a-brac and cap ‘n’ collar homology 1 (BACH1), has been labeled an essential factor involved in RCC progression in vivo [32]. BACH1-mediated RCC evolution contributes to increased invasion and migration abilities, activation of epithelial–mesenchymal transition, dysregulation of inflammatory responses, immunomodulation, cytoprotection, the anti-inflammatory response, angiogenesis, mTOR, increased tumor metabolism, and the metastatic potential [23,24,25,32,33].

A recently detected set of genes associated with cellular metabolism has been identified as involved in ccRCC distant metastasis [2,26]. Metabolic alterations (hyperglycemia, hyperlipidemia), anomalies in calcium-dependent signaling, mutational changes, ROS-induced damage, heat shock, and exposure to toxins/medications [14,26,27] can lead to the activation of cellular stress, called the unfolded protein response [14].

Genetic and epigenetic changes play a crucial role in altering signaling networks, pathogenesis, and the prognosis of RCC [23]. Genes whose alteration leads to RCC formation are tumor suppressors (von Hippel–Lindau (VHL), tuberous sclerosis complex (TSC)) or oncogenes (receptor tyrosine kinase-MET, specific pRCC) [1]. Commonly mutated genes in ccRCC include the tumor suppressor VHL gene, Polybromo 1 (PBRM1), BRCA1-associated protein 1 (BAP1), SET domain containing 2 (SETD2), lysine demethylase (UTX), AT-rich interaction domain 1A (ARID1A), and lysine demethylase 5a (KDM5a), which induce upregulation of hypoxia in the tumor microenvironment, alter metabolism, and enhance cell proliferation, angiogenesis, migration, invasion, and RCC metastasis [2,14,34]. Other evidence has shown that a TME rich in inflammatory cells favors tumor proliferation, survival, and migration [35].

Tumor angiogenesis, a crucial process in the pathogenesis of RCC, facilitates metastasis by forming abnormal neovessels that allow tumor cells to enter the bloodstream. These aberrant vascular networks affect the TME and create a hostile microenvironment characterized by acidity and hypoxia. It is known that the constitutive upregulation of growth factors (vascular endothelial growth factor (VEGF), platelet-derived growth factor (PDGF), insulin-like growth factor 2 (IGF2), erythropoietin) and the induction of hypoxia-inducible factor 1α (HIFα) contribute to RCC development by stimulating vascularization and aberrant cell proliferation and inhibiting apoptosis [36]. Additionally, branched-chain keto-acid dehydrogenase kinase upregulation in ccRCC, extracellular vesicles, exosomes, cancer stem cell reactivation, long non-coding RNA (lncRNA), microRNA (miRNA), and mRNA enable communication between cells in the TME, indirectly or directly contributing to tumor development, angiogenesis, invasion, and RCC metastasis [2,30,37,38]. Branched-chain keto-acid dehydrogenase kinase is the rate-limiting enzyme of branched-chain amino acid metabolism, upregulated in ccRCC [38]. The branched-chain keto-acid dehydrogenase kinase/exosome-lncRNA/miRNA/mRNA/extracellular vesicle/VE-cadherin axis regulates intercellular communication in ccRCC and plays a critical role in renal cancer metastasis [30,38,39,40]. Current treatments for advanced RCC aim to obstruct the growth of new blood vessels or angiogenesis and target tumor cells.

Another important player involved in the pathogenesis of RCC is mitochondria. Mitochondrial quality influences RCC progression. Mitochondria are functionally versatile, participating in processes such as fusion, fission, and controlled turnover, allowing rapid adaptation to metabolic conditions and ensuring reprogrammed cellular metabolism unfolds. Recently, the hypothesis of horizontal transfer of mitochondria between tumor and non-tumor cells in the TME has been proposed. This transfer may be a strategy cancer cells adopt to counteract mitochondrial dysfunction and meet high metabolic demands [35]. Mitochondrial dysfunction can promote RCC and metastases (ccRCC and chRCC) by altering mitochondrial genes related to oxidative stress, genetic instability, excessive intracellular ROS generation, inactivation of key enzymes of energy metabolism, intracellular macromolecule oxidation, autophagy, damage, and ferroptosis/cell death [2,12,13,14,35,41].

Multiple studies have explored the role of inflammation in promoting tumorigenesis, immune evasion, poor differentiation, and progression of urological cancers [14,15,42,43]. Cancer-related chronic inflammation leads to unlimited proliferative potential, independence from growth factors, enhanced angiogenesis, metastasis, evasion of apoptosis, and resistance to growth inhibition [35]. Stimulation of production and secretion of inflammatory mediators (IL-6, TNF-α, IL-1β), amplification of associated signaling pathways (JNK, NF-kB), immune cell infiltration, and UPR (by activating IRE1α, PERK, and ATF6) can contribute to maintaining an inflammatory environment, which will facilitate RCC proliferation, angiogenesis, invasion, and progression [14,43,44].

**Figure 2:**
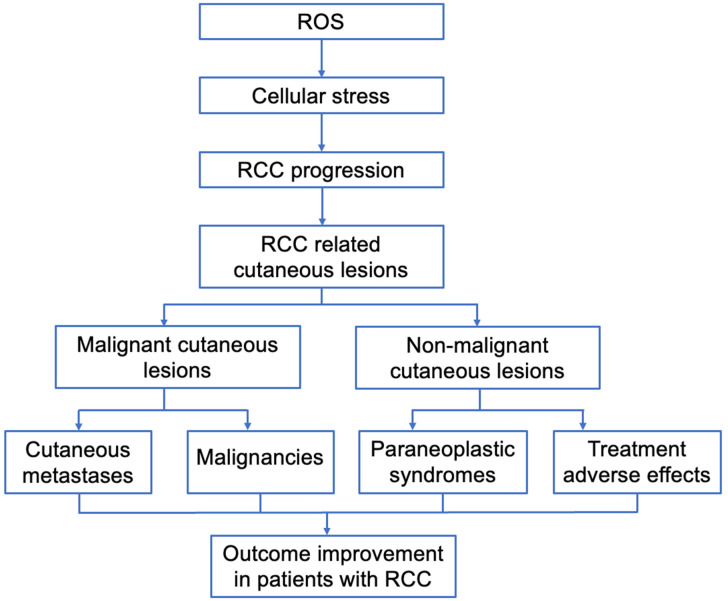
Dermatological conditions in RCC patients (ROS—reactive oxygen species; RCC—renal cell carcinoma).

The specific composition of the TME can influence tumor progression and evasion, and its progressive dysfunction has been correlated with the disease stage in ccRCC patients [34]. Standard TME regulation mechanisms are disrupted by aberrant microRNA expression in RCC [45,46], reciprocal interactions between neoplastic, stromal, and inflammatory cells [43], excessive ROS [2], aberrant microRNA expression in RCC [45,46], and the self-renewal capacity and clonogenic multipotency of CD133+/CD24+ cells in ccRCC specimens (referred to as “cancer stem cells”) [17,18]. Potential damage can be avoided through the horizontal transfer of mitochondrial DNA and the upregulation of detoxification systems [35]. Current findings support the concept that cellular stress orchestrates RCC progression (Figure 2).

## 3. Cutaneous Events in RCC

RCC can trigger immunologic responses that influence the disease’s natural course and clinical presentation. Recognizing atypical manifestations of the disease, such as cutaneous symptoms, is crucial. It is known that RCC is characterized by anarchic cell proliferation, which escapes the control of regulatory mechanisms and results in the appearance of distant metastases. The skin often signals systemic changes. Secondary cutaneous events may occur concurrently with RCC, disappear after tumor removal, and increase with tumor recurrence or metastatic disease [5,47,48,49]. The skin can be directly or indirectly involved in malignant kidney conditions [5].

### 3.1. Malignant Cutaneous Lesions Associated with RCC

The presence of neoplastic cells in skin lesions is represented by RCC metastases or other malignant entities developed synchronously or metachronously with RCC. Unlike healthy cells in the body, cancer cells have the unique property of multiplying uncontrollably, even outside their place of origin. Tumor cells do not change; they maintain their structure but migrate to other body parts [49].

#### 3.1.1. Distant Metastasis of RCC to the Skin

Highly angioinvasive tumors, such as RCC, tend to generate metastases with an unpredictable pattern of distribution, far from the primary tumor. RCC is the most common genitourinary cancer, which metastasizes to the skin and represents 6.8% of cutaneous metastases of these malignancies [50]. Communication between the kidney and the skin is bidirectional. The reverse situation has been also reported, in which primary cutaneous neuroendocrine or Merkel cell carcinoma develops renal metastases [51].

Cutaneous metastatic lesions may be the primary presentation of the disease at the time of diagnosis, and 20–50% of RCC patients may develop metastases after nephrectomy [28,52]. Sometimes, metastatic cells can remain dormant for decades, leading to late manifestations [53,54,55].

The spread of a primary tumor to the skin is attributed to several mechanisms: ectopic hormone production (such as erythropoietin, parathyroid hormone-associated protein, gonadotropins, human placental lactogen, an adrenocorticotropic hormone-like substance, renin), the presence of tumor-associated extracellular microvesicles, the angiogenic switch regulating the ratio between the proliferative and resting phases of the cell, the tumor microenvironment, genetic and molecular factors, and the intrinsic capacity of malignant cells to evade the immune response [55].

The cutaneous distribution mechanism depends on the spread route through lymph, blood, peritoneum, the iatrogenic route, or contiguity [55,56]. RCC tends to spread hematogenously from the renal vein to the vena cava and vertebral veins to reach the central nervous system or to the spermatic or ovarian vein to reach the pelvic organs. Additionally, RCC growth is associated with VEGF, explaining its ability to disseminate widely and form highly vascularized dermal metastases [56].

Cutaneous metastases are not frequent, and the incidence and clinical manifestations are poorly established in the literature (Table 1). Various reports have described the spread of RCC to the head, neck, trunk, abdomen, testicles, penis, and extremities [50,56,57,58,59,60,61,62,63,64]. The most common sites for cutaneous metastasis in RCC have been the trunk (40%), scalp (25%), and face (9%) of all metastatic lesions [65]. Other unusual reported sites include the chin, preauricular area, tongue, thigh, fingers, and axilla [50,66,67,68]. Metastases to distal extremities are rare and represent less than 0.2% of all RCC metastatic lesions. Cutaneous metastases of the fingers present as erythematous pustules on the fingertips, resembling a pyogenic granuloma [65]. There are a few reports in the literature presenting an unusual pattern of cutaneous metastatic RCC involving the testicles and the penis [56,57,58]. The testicular localization of metastatic RCC was identified following radical nephrectomy for localized disease [57]. The tendency for RCC metastases to occur in the testicles (ipsilateral, contralateral, bilateral) may be related to preferential spread pathways between the kidney and testicles, including retrograde venous spread through the spermatic vein, Baston’s venous complex, and iatrogenic spread of cancer cells to the testicles [58]. Testicular metastases in RCC patients suggest a late stage of the disease and do not present typical systemic or specific symptoms except for swelling and enlargement of the affected testicle [69]. Metastatic RCC involving the penis has been described as a lesion resembling a pyogenic granuloma with painful, persistent penile nodules. Identifying the spread of a primary tumor to atypical skin areas can indicate disease progression and may suggest an overall poor prognosis [56].

Cutaneous metastases originating from ccRCC are positive for CAM5.2, EMA, CD10, RCC-Ma, vimentin, and S100 and negative for Melan-A, TTF-1, CK7, and CK20 [70].

#### 3.1.2. Metachronous and Synchronous Skin Malignancies Associated with RCC

Multiple metachronous or synchronous malignancies have been reported in the same patient with RCC, including lymphoid malignancies (chronic lymphocytic lymphoma, chronic lymphocytic leukemia, Hodgkin lymphoma, mycosis fungoides), and cutaneous malignancies such as basal cell carcinoma, squamous cell carcinoma, and melanoma [14,35,49] (Table 1).

The incidence of chronic lymphocytic lymphoma and ccRCC occurring in the same patient is higher than in the general population. Various explanatory hypotheses for this co-occurrence include the development of the second malignancy related to treatment, immunomodulatory mechanisms, viral etiology, cytokine release from a tumor, or genetic mutations [71]. Although reports of the coexistence of RCC with chronic lymphocytic leukemia are rare, the unusual occurrence of RCC metastases in the neck region has been documented in the context of chronic lymphocytic leukemia progression many years after curative nephrectomy [72]. On the other hand, cutaneous T-cell lymphomas (non-Hodgkin lymphomas, mycosis fungoides, Sezary syndrome) are aggressive clinical conditions that occur in association with ccRCC. The relationship between RCC and lymphoproliferative malignancies in the same individual also suggests a familial predisposition [73]. Other authors have reported non-Hodgkin lymphoma occurring in association with oncocytoma and RCC in the same patient or being incidentally discovered after radical nephrectomy for RCC [74,75]. It is acknowledged that this coexistence is due to decreased cellular immunity, immune surveillance, or genetic factors [73,76,77]. These findings indicate an increased risk of simultaneous occurrence of two different malignancies in the same patient.

Based on the available reports to date mentioning the strong association between cutaneous cancer (melanoma, squamous cell carcinoma, eccrine porocarcinoma, Kaposi sarcoma) and RCC, specific characteristics emerge, such as a high risk of developing secondary malignancy, and the simultaneous presence of multiple malignancies in the same patient, with different locations, morphologies, histology, etiologies, and pathogenesis [78,79,80,81,82]. Regarding the coexistence of cutaneous melanoma and RCC, it has been noted that these neoplasms predominantly occur in certain families and have an inherited pattern of cancer predisposition [78,79]. Moreover, eccrine porocarcinoma, an isolated malignancy from eccrine sweat glands, can coexist with urological cancers, such as RCC [80]. A clinical case of a patient with advanced RCC and squamous cell carcinoma of the zygomatic region showed a durable response to immunotherapy [81]. The simultaneous occurrence of Kaposi sarcoma (classic, endemic, associated with human immunodeficiency syndrome or transplantation) and RCC may be triggered by a common angiogenic factor, VEGF [82]. Multiple primary malignant neoplasms represent an unusual phenomenon. Multiple metachronous or synchronous malignancies diagnosed in the same patient have also been represented by RCC, lung cancer, basal cell carcinoma, and melanoma [49]. RCC confers a risk of 0.6% of developing a second neoplasm [79].

There are also situations where RCC causes the development of amyloidosis. Amyloidosis involves the deposition of amyloid protein in tissues and is the most severe systemic complication of any chronic inflammatory condition. The increase in serum amyloid A is an acute-phase response stimulated by high levels of IL-6, IL-1, and TNF-alpha derived from RCC [83,84]. These findings highlight the potential role of immune system dysregulation in patients with multiple malignancies, whereby the first malignancy predisposes the patient to the second malignancy through an immunomodulatory effect. Therefore, RCC can be a second malignant neoplasm in lymphomas or mycosis fungoides and a primary malignancy in amyloidosis [71,76,77,83,84,85].

### 3.2. Paraneoplastic Syndromes in RCC 

Indirect involvement of the skin in the development of paraneoplastic syndromes is caused by the release of peptides, hormones, or cytokines secreted from tumor tissue or cross-reactivity between tumor cells and normal tissues. Paraneoplastic syndromes are observed in approximately 40% of RCC cases [86]. The most common cutaneous manifestations associated with RCC are papulosquamous, erythematous, or bullous lesions (Table 1).

Rarely, RCC is associated with acanthosis nigricans. Acanthosis nigricans is a skin condition characterized by hyperpigmentation, hyperkeratosis, and papillomatous hyperplasia. Several clinical variants of acanthosis nigricans are known. A paraneoplastic variant can often be differentiated from other variants of acanthosis nigricans by its extensive lesions on the skin and mucous membranes, rapid progression, and onset usually in middle age. The coexistence of acanthosis nigricans and RCC is explained by the hyperstimulation of keratinocytes induced by the action of excess TNF-α on VEGFR [87,88].

The Leser–Trélat sign has occasionally been identified in renal pathology (RCC, chronic kidney disease). It is a paraneoplastic skin syndrome characterized by the eruptive appearance of multiple seborrheic keratoses, caused by clonal expansion of immature keratinocytes [89,90,91]. It has been hypothesized that the sudden eruptions are the result of cytokines and growth factors produced by neoplastic cells [91]. Intense immunoexpression of EGFR, EGF, TGF-α, or amphiregulin affects the development of seborrheic keratoses. Overexpression of EGFR could be one of the mechanisms responsible for the sudden eruption of multiple seborrheic keratoses [89,90].

The sudden appearance of diffuse palmoplantar keratoderma, accompanied by itching in patients with RCC, which does not resolve with topical keratolytic agents and emollients, can be categorized as a paraneoplasia [92].

Spiny keratoderma, associated with RCC, is classified as unusual. It is a rare palmoplantar condition characterized by multiple foci of hyperkeratosis, orthokeratosis, and occasionally parakeratosis, acantholysis, and dilated, delimited dermal capillaries present on the palms and soles. The literature reports that it is associated with various underlying conditions, including RCC [93,94].

Scrofuloderma with skin involvement is suggestive of patients with compromised cellular immunity. It has been documented in metastatic RCC [95]. Caused by Mycobacterium tuberculosis or related strains, scrofuloderma presents a wide range of clinical manifestations, with delayed diagnosis, mimicking other chronic dermatoses. The clinical picture is characterized by subcutaneous, painless nodules with slow growth, evolving into ulcers, fistulas with purulent discharge, atrophic sequelae, or spontaneous healing [95].

Acquired ichthyosis and erythema gyratum repens have been reported as two paraneoplastic skin disorders present in the same patient with transitional cell carcinoma [96]. From case reports and observational studies, it has been noted that erythema gyratum repens precedes the diagnosis of malignancy, while acquired ichthyosis occurs before, simultaneously, or after the onset of malignancy [97]. The appearance of two paraneoplastic skin disorders in the same patient can be explained by the secretion of TNF-alpha by tumor cells, which has been shown to be a mitogen for keratinocytes [96,98].

Urticaria and angioedema appear as dermatologic manifestations with atypical characteristics present in RCC [99,100]. Chrophobe RCC has been associated with mild urticaria, severe angioedema, progressive fatigue, and adynamia. After radical nephroureterectomy and treatment with antihistamines, a rapid improvement in urticaria and facial angioedema was observed [100]. In the case of an underlying RCC, secondary angioedema, accompanied by progressive swelling of the face, lips, and tongue, has been reported [100].

The incidence of RCC in patients with psoriasis is rare. In recent decades, concerns have been expressed regarding the role of systemic inflammation in promoting various organ neoplasms [101]. It is noteworthy that spontaneous regression of RCC metastases after nephrectomy involves activation of the host immune system [102].

Idiopathic inflammatory myopathies associated with RCC are events recently described in the literature. Idiopathic inflammatory myopathies include conditions such as dermatomyositis, polymyositis, sporadic inclusion body myositis, and immune-mediated necrotizing myopathy [103,104]. These idiopathic inflammatory myopathies, characterized by muscle weakness, pain, and rash, are sometimes associated with an underlying malignancy and are described as paraneoplastic syndromes [105]. Renal tumors have been rarely identified in dermatomyositis and have been published as case reports [103,105,106,107,108]. A brief analysis of these reports (five cases) highlighted: an average age over 60 years, a male-to-female ratio of 2:3, the presence of urinary symptoms in only one case, a diagnosis of a paraneoplastic condition that preceded detection of advanced-stage RCC, vulnerability to infections caused by immunosuppressive therapy applied, and abnormalities in serum muscle enzymes (creatine kinase, aldolase) [103,105,106,107,108]. An occasional event is amyopathic dermatomyositis, reported in a patient with RCC presenting with hematuria [105].

The multiorgan autoimmune paraneoplastic syndrome (PAMS) has been reported in RCC. Paraneoplastic pemphigus, more recently termed PAMS, includes several clinical variants: pemphigus-like, pemphigoid-like, erythema multiforme-like, graft-versus-host disease-like, and lichen planus-like [72,109,110]. The pathogenesis of PAMS may be due to either the antitumor immune response, dysregulated cytokine production, or autoantibodies derived from tumor-origin B cells [110]. Cutaneous eruptions and recalcitrant stomatitis, early manifestations in PAMS, allow for the detection of an incipient malignancy [109,110].

The association between linear IgA dermatosis and malignancy suggests a causal relationship. Linear IgA dermatosis, characterized by subepidermal blisters and IgA deposition in the basement membrane, has been documented in advanced RCC. The data presented in the literature support the concept that linear IgA bullous dermatosis may be a paraneoplastic syndrome of solid tumors [111,112].

The likely association between bulky RCC and hypertrophic lichen planus may appear as a paraneoplastic manifestation [113].

Paraneoplastic vasculitis is an immune-mediated paraneoplastic syndrome detected in patients with rapidly growing RCC. Paraneoplastic vasculitis develops in cancer patients, with a prevalence of 0.01–1.0% in localized cancer and up to 8% in metastatic cancer. Among various types of vasculitis, leukocytoclastic vasculitis has been the most common form of vasculitis in advanced RCC. Based on the literature data, it can be appreciated that the skin is most affected by leukocytoclastic purpura, especially at the limb level. It has not been associated with patient outcomes, but it is suggested to be a negative prognostic factor [114]. Leukocytoclastic vasculitis can also be induced by immune checkpoint inhibitors used in treating patients with advanced RCC [115].

Multicentric reticulohistiocytosis is a rare non-Langerhans cell histiocytosis associated with RCC. Extensive joint and skin involvement required treatment with infliximab and methotrexate, leading to the resolution of symptoms and cutaneous manifestations [116].

Pyoderma gangrenosum has been associated with ccRCC. It presents as a painful, recurrent, fibrinous, and necrotic ulcer that rapidly grows. It has numerous neovessels and a polymorphic inflammatory infiltrate abundant in neutrophils that extend into the subcutaneous tissue without signs of vasculitis [117]. Preoperatively diagnosed pyoderma gangrenosum has also been described in a patient with chronic lymphocytic leukemia and RCC [118].

Ongoing studies have demonstrated associations between inherited cancer susceptibility syndromes, RCC, and skin [119,120]. Patients diagnosed with hereditary leiomyomatosis and RCC-associated leiomyomatosis present with a fumarate hydratase gene mutation. This mutation leads to intracellular fumarate accumulation, which mediates proteomic and epigenetic events, ultimately leading to HIF-1 activation. Cutaneous manifestations include multiple cutaneous piloleiomyomas, presenting as firm, reddish-colored papules or nodules [119,120]. Birt–Hogg–Dubé syndrome is a rare autosomal dominant inherited disorder associated with ccRCC and chRCC, caused by point mutations and rearrangements of the folliculin gene. This gene functions as a tumor suppressor and is involved in cell–cell adhesion, negative regulation of ribosomal RNA synthesis, and modulation of the mTOR pathway. This syndrome is characterized by the development of multiple renal tumors, facial fibrofolliculomas, and pulmonary cysts [119].

**Table 1:** Clinical and histological findings in metastases of ccRCC, skin malignancies, and paraneoplastic syndrome related to RCC.

Cutaneous Manifestations	Clinical Findings	Histological Findings	References	
Metastases of ccRCC	A solid, yellowish lesion with internal necrosis and hemorrhage	Cells with cytoplasm rich in lipids and glycogen, hypervascularized	[56,57,58,59,60,61,62,63,64]	
Skin malignancies				
Mycosis fungoides	Papules, tumors, ulcers	Infiltrates malignant T cells with irregular cerebriform nuclei, localized in the dermis	[71,72,73,74,75,76,77,83,84,85]	
Basal cell carcinoma	Pearly nodule with arborizing vessels on its surface +/− ulceration	Basaloid palisading epithelium, basal cells with hyperchromatic nuclei, apoptotic cells	[49]	
Squamous cell carcinoma	Keratinous plaque/nodule with irregular margins that can ulcerate	Nests of atypical squamous epithelial cells in the dermis	[81]	
Melanoma	Brittle nodule, which bleeds easily, with variable dimensions and chromatic polymorphism	Nests of melanocytes in lymphovascular spaces, with atypical mitoses, atypical nuclei, and increased apoptosis	[78,79]	
Paraneoplastic syndromes				
Acanthosis nigricans	Hyperpigmented, infiltrated, keratotic lesions in intertriginous areas	Hyperkeratosis, papillomatosis, acanthosis with thickening of the spinous layer of the epidermis	[87,91]	
Leser–Trélat syndrome	Papular, verrucous, pruritic lesions	Abundant inflammatory infiltrate	[92]	
Palmoplantar keratoderma	Excessive thickening of the epidermis	Hyperkeratosis	[93,94]	
Spiny keratoderma	Keratotic patches	Keratotic plugs	[93,94]	
Scrofuloderma	Nodules, ulcers, purulent discharge fistulas	Inflammatory granulomatous infiltrate, necrosis, acid-fast bacilli	[95]	
Acquired ichthyosis	Grayish-brown scales	Focal parakeratosis, thickened granular layer, acanthosis, hyperplasia	[97,98]	
Erythema gyratum repens	Serpinginous, polycyclic, and pruritic erythema	Hyperkeratosis, parakeratosis, acanthosis, spongiosis, lymphohistiocytic infiltrate	[96]	
Urticaria	Multiple erythematous and edematous lesions, accompanied by intense itching	Dermal edema, perivascular inflammatory infiltrate	[99,100]	
Psoriasis	Well-demarcated erythematous and scaly plaques	Parakeratosis, regular acanthosis of the epidermis, Munro microabcesses, lymphocytic dermal infiltrate	[101,102]	
Idiopathic inflammatory myopathies	Muscle weakness, pain, and skin rash	Hyperkeratosis, parakeratosis, acanthosis, and spongiosis with perivascular mononuclear inflammatory infiltrate	[103,105,106,107,108]	
PAMS	Erythroderma and refractory stomatitis	Lichenoid infiltrates with interface dermatitis, necrotic keratinocytes, and strong epidermotropism	[72,109,110]	
Linear IgA dermatosis	Clear or hemorrhagic vesicles/bullae on normal/erythematous skin	Suprabasal acantholysis and keratinocyte necrosis IgA deposition along the basement membrane	[111,112]	
Paraneoplastic vasculitis	Palpable purpura	Vascular inflammation and blood vessel involvement	[115]	
Multicentric reticulohistiocytosis	Papules, nodules, and rapidly evolving arthritis that can affect any joint	Histiocytes with abundant cytoplasm, mild nuclear atypia	[116]	
Pyoderma gangrenosum	Painful, recurrent, fibrinous, and necrotic ulcer with rapid growth	Necrotic dermal vessels, and a neutrophilic inflammatory infiltrate	[117]	

RCC—renal cell carcinoma, PAMS—multiorgan autoimmune paraneoplastic syndrome

### 3.3. Adverse Cutaneous Drug Events in RCC Patients

It is noteworthy that RCC presents a multitude of treatment challenges attributable to patients’ delayed presentation for diagnosis. The earlier a cancer is diagnosed during its progression, the more successfully it can be treated, and the patient’s quality of life is not as significantly impacted. Therapeutic options for renal cancer are numerous, including surgery, radiotherapy, systemic treatments (targeted oral therapies, immunotherapy with immune checkpoint inhibitors, and immunomodulators such as interleukins and interferons), or combinations thereof [18].

Conventional chemotherapy and radiotherapy are ineffective in patients with metastatic ccRCC. In recent years, immunotherapy has contributed significantly to cancer treatment. Monotherapy with immune checkpoint inhibitors or immune-based combination therapies is associated with improved survival, regardless of performance status [28]. Drug-induced cutaneous adverse reactions encompass a wide spectrum of skin manifestations, ranging from mild or moderate events to severe and life-threatening conditions [121,122,123,124,125]. Cutaneous manifestations are one of the most common immune-related adverse events, presenting with a variety of manifestations: superficial perivascular dermatitis, interface dermatitis, psoriasis, acantholytic dermatitis, acute generalized exanthematous pustulosis, and erythema nodosum-like panniculitis [121,123,124,125].

Chemotherapy administration can lead to many cutaneous findings, ranging from allergic reactions to infectious complications caused by disturbed immunity. The most common cutaneous reactions caused by chemotherapy are erythema multiforme, chemotherapy-induced toxic erythema, and toxic epidermal necrolysis [126]. Adverse cutaneous findings occurring during RCC treatment could result from direct cutaneous toxicity or drug hypersensitivity reactions [126,127,128].

Immune checkpoint inhibitors represent a relatively new class of drugs whose administration has been approved for RCC. A comprehensive analysis based on genes associated with the immune response (IRG: CLDN4, SEMA3G, CAT, and UCN) and the presence of tumor mutations has shown that ccRCC patients demonstrate increased sensitivity to immune checkpoint therapy (PD-1, CTLA-4, IL-6, and LAG3) [129]. Adverse events are fairly common, with the skin being the most frequently involved organ [121]. Potential pathogenic mechanisms include drug-induced neoantigen formation, unmasking of usually hidden autoantigens, and generalized activation of CD4+ and CD8+ T cells, leading to diffuse keratinocyte apoptosis [121]. Cutaneous immune-related adverse events induced by immune checkpoint inhibitors can be grouped as follows: (a) inflammatory dermatoses (maculopapular eruptions) and papulosquamous disorders (pityriasis rosea, pityriasis lichenoides, pityriasis rubra pilaris, lichenoid and psoriasiform lesions); (b) immune bullous dermatoses (bullous pemphigoid, linear IgA dermatosis); (c) melanocyte alterations (vitiligo-like skin depigmentation, tumoral melanosis and regression of melanocytic nevi, poliosis, and eyebrow or eyelash depigmentation); (d) keratinocyte alterations (benign, precancerous, and cancerous keratinocytic lesions, mainly on photodamaged skin, seborrheic keratoses, actinic keratoses, keratoacanthomas, basal or squamous cell carcinomas); (e) hair abnormalities (non-scarring alopecia, partial or diffuse alopecia areata, hypotrichosis, vitiligo, universal alopecia, hair texture changes); (f) nail involvement (nail dystrophy, mostly with psoriasiform or lichenoid features, onychomadesis and proximal onychoschizia, diffuse onycholysis and paronychia); (g) oral involvement (mucositis, gingivitis, xerostomia, dysgeusia, Sjogren syndrome); and (h) rare reactions (photosensitivity, dermatomyositis, panniculitis, granulomatous dermatitis, lymphomatoid or eosinophilic cutaneous toxicity, acute generalized exanthematous pustulosis, neutrophilic dermatoses, toxic epidermal necrolysis) [22,121,130,131,132].

Multitargeted kinase inhibitor therapy, a strategy adopted for RCC patients, has led to hand–foot skin reactions, characterized by well-defined erythematous papules and plaques, tender gray vesicles or hyperkeratotic formations, epidermal acanthosis, papillomatosis, parakeratosis, and scattered dyskeratotic cells [122]. Other skin manifestations include angular cheilitis, seborrheic dermatitis, and perianal dermatitis. The most relevant histopathological findings of hand–foot skin reactions include vacuolar degeneration of keratinocytes, the presence of intracytoplasmic eosinophilic bodies, and intraepidermal vesicles in the Malpighian layer [122]. Sorafenib, a multikinase inhibitor, induces acneiform eruptions, erythema, rash/desquamation, hand–foot syndrome, alopecia, pruritus, xerosis, bullous lesions, nail changes, facial erythema, subungual hemorrhages, erythema multiforme, and keratoacanthomas [123]. A post-nephrectomy RCC patient developed hand–foot syndrome with each cycle of targeted therapy with sorafenib [123]. Sunitinib, a novel multitargeted kinase inhibitor (inhibitor of tumor angiogenesis, selective inhibitor of VEGFR-2, VEGFR-3, PDGFR), produces hand–foot skin reactions and alopecia. RCC patients taking sorafenib or sunitinib should be informed about the potential development of these conditions to minimize distress [122,123,124,131].

Anti-angiogenic drugs are currently the primary treatment option for metastatic ccRCC. The main dermatological adverse reactions during pembrolizumab/axitinib treatment have been rashes, blisters, erythrodysesthesia syndrome, and severe skin reactions. Stasis dermatitis may affect the onset and worsening of immune-related dermatitis [125,133]. Recent randomized controlled phase II and III trials conducted in patients with solid malignancies have shown that anti-VEGF agents have been associated with an increased risk of cardiovascular events. In contrast, combining anti-VEGF with immune checkpoint inhibitors has not demonstrated a significantly increased risk of cardiotoxicity [134].

A comprehensive approach and early recognition of cutaneous events attributed to RCC guide us in considering the skin as a surface reflective of the signs and symptoms that occur at distant sites of an underlying malignancy, such as RCC.

## 4. Conclusions

Presenting the main cutaneous manifestations associated with renal malignancies will enhance the understanding of the metabolic basis of RCC. This could provide insights that are helpful for developing an effective therapy for patients with this disease. The prognosis of patients varies depending on the stage at which the condition was diagnosed and the treatment administered. Additionally, new opportunities for molecular imaging are being developed to diagnose RCC preoperatively and to limit postoperative renal function loss. The earlier a cancer is diagnosed during its progression, the more successfully it can be treated, and the patient’s quality of life is not as significantly impacted.

This article presents information supporting the hypothesis that secondary cutaneous processes attributed to RCC develop from a coordinated interaction between inflammatory, metabolic, and proliferative factors and cellular stress responses. From the documented data, RCC is the most common primary urological malignancy with metastatic potential for the skin. This paper has represented the first comprehensive review of the available information evaluating the cellular stress–RCC–cutaneous secondary events axis in RCC patients. Based on this interaction, systemic events associated with skin involvement may reflect direct metastatic spread, indirect manifestations of RCC, or treatment consequences. These data will aid in devising better strategies for preventing RCC and managing RCC patients.
